# Middle East Respiratory Syndrome Coronavirus Seropositivity in Camel Handlers and Their Families, Pakistan

**DOI:** 10.3201/eid2512.191169

**Published:** 2019-12

**Authors:** Jian Zheng, Sohail Hassan, Abdulaziz N. Alagaili, Abeer N. Alshukairi, Nabil M.S. Amor, Nadia Mukhtar, Iqra Maleeha Nazeer, Zarfishan Tahir, Nadeem Akhter, Stanley Perlman, Tahir Yaqub

**Affiliations:** University of Iowa, Iowa City, Iowa, USA (J. Zheng, S. Perlman);; University of Veterinary and Animal Sciences, Lahore, Pakistan (S. Hassan, I.M. Nazeer, T. Yaqub);; King Saud University, Riyadh, Saudi Arabia (A.N. Alagaili, N.M.S. Amor);; King Faisal Specialist Hospital and Research Centre, Jeddah, Saudi Arabia (A.N. Alshukairi);; Government of Punjab, Lahore (N. Mukhtar, Z. Tahir, N. Akhter);; The First Affiliated Hospital of Guangzhou Medical University, Guangzhou, China (S. Perlman)

**Keywords:** Middle East respiratory syndrome, coronavirus, pneumonia, Pakistan, camel handlers, MERS-CoV, viruses, respiratory infections, zoonoses

## Abstract

A high percentage of camel handlers in Saudi Arabia are seropositive for Middle East respiratory syndrome coronavirus. We found that 12/100 camel handlers and their family members in Pakistan, a country with extensive camel MERS-CoV infection, were seropositive, indicating that MERS-CoV infection of these populations extends beyond the Arabian Peninsula.

Middle East respiratory syndrome (MERS) coronavirus (MERS-CoV), identified in 2012, causes a highly lethal pneumonia with a 34.5% mortality rate (https://www.who.int/emergencies/mers-cov). As of July 31, 2019, a total of 2,458 cases and 848 deaths have been reported to the World Health Organization, with all cases in the Middle East or in travelers from this region or their contacts (*1*). MERS cases fall into 2 categories, primary and secondary. Secondary cases, which result most commonly from human-to-human transmission in hospitals, were most prominent during the early years of the outbreak. However, as stringent infection control measures have been followed more closely, a greater proportion of cases are classified as primary. Camels are believed to be the zoonotic source for primary infections, but a large proportion of patients describe no camel contact, raising the question of how they acquired the disease (*2*). 

To determine the source of the infection, several studies have focused on a potential role in transmission for camel handlers. These reports indicate that the percentage of MERS-CoV–immune camel handlers is much greater than in the general population of Saudi Arabia, the country with the largest number of MERS cases. These studies have reported that 3%–67% of camel handlers in this country are MERS-CoV exposed, compared with 0.15% of the general population (*3*–*5*). In Saudi Arabia, much of camel farming is labor intensive, and many camel owners hire camel handlers, generally from outside of the country, to tend to them (*6*). To determine the generalizability of these observations, we tested blood samples from 100 camel handlers and their families in the Cholistan desert in Punjab, Pakistan, a country with no reported human MERS (*7*). 

## The Study

We chose Cholistan as the study site because it is the most important region of Pakistan for the camel industry, and handlers and their families are in close contact with dromedaries. We engaged study participants in the Bahawalnagar and Bahawalpur districts, located in southern Punjab Province, Pakistan. The Institutional Ethical Review Board (IERB) of the Institute of Public Health, Government of Punjab, Lahore, Pakistan, approved the study. We obtained written informed consent from all study participants.

Camel handlers in Cholistan differ from those in Saudi Arabia in that they own their camels, along with cows, goats, and sheep, and they and their families take care of these animals. Both men and women are responsible for grazing, feeding, milking, and waste disposal. In addition, they live in close proximity to camels and share similar water sources (*8*–*10*). Camel handlers in Cholistan are either nomadic, seminomadic, or sedentary, with varying degrees of exposure to camels. Nomads live with their camels in the desert and migrate throughout Cholistan, whereas seminomads tend to live at a base camp and migrate depending on availability of fodder and water. Nomadic camel handlers and their families have the highest exposure to camels, whereas sedentary ones have the least exposure.

During 2017–2018, we obtained blood samples from 100 participants from nomadic, seminomadic, and sedentary populations. The age range was 8–76 years (average 30.1 years). We obtained demographic and clinical information at sampling by written questionnaire, including participant age, lifestyle (nomadic, seminomadic, sedentary), role in family (husband, wife, child, etc.), underlying medical conditions, numbers of camels owned, history of tobacco use (smoking or chewing), and consumption of camel products (milk) (Table 1; Appendix Table). We transported samples to the microbiology department at the University of Veterinary and Animal Sciences (Lahore, Punjab, Pakistan). We prepared serum samples, stored them at −80°C, and shipped them to the University of Iowa (Iowa City, Iowa, USA) for analysis. 

**Table 1:** Characteristics of participants in study of Middle East respiratory syndrome coronavirus seropositivity in camel handlers and their families, Pakistan

Characteristic	No. (%) participants
Lifestyle	
Sedentary	10 (10)
Seminomadic	64 (64)
Nomadic	26 (26)
Concurrent conditions	21 (21)
Consumption of unpasteurized camel milk	98 (98)
Tobacco use	38 (38)

We tested all the samples for MERS-CoV–specific antibodies by ELISA and 50% reduction plaque-reduction neutralization test (PRNT_50_). Of 91 participants examined by a commercially available ELISA, 49 were positive for MERS-CoV–specific antibody. Twelve had PRNT_50_ titers >1:20 and were considered positive; of these, 5 were also positive by ELISA. In addition, 10/12 were positive by immunofluorescence assay. Of the 12 PRNT_50_-positive participants, 3 were women and 1 was an 8-year-old child (Table 2). 

**Table 2:** Characteristics of camel handlers and their families positive for Middle East respiratory syndrome coronavirus in study in Pakistan*

Patient no.	Family no.	Age, y/ex	Camel contact†	Smoking	Concurrent conditions	Lifestyle	PRNT_50_	ELISA result/ value‡	IFA result/§
SH94	F2	20/M	Direct/daily	Yes	None	Nomadic	211	–/0.78	+/1:80
SH85	F2	21/M	Direct/daily	Yes	None	Nomadic	32	+/1.51	+/1:40
SH100	F1	8/M	Direct/daily	No	None	Nomadic	72	–/0.64	+/1:40
SH71	F9	35/F	Indirect	No	HPT, renal and respiratory disease	Seminomadic	33	Borderline/​.99	+/1:20
SH74	F9	40/F	Indirect	No	HPT	Seminomadic	40	+/2.18	+/1:80
SH63	F13	35/F	Direct/monthly	No	None	Seminomadic	27	Borderline/​0.92	+/1:10
SH57	F14	20/M	Direct	No	None	Seminomadic	51	+/3.11	+/1:80
SH58	F16	28/M	Direct	Yes	None	Seminomadic	68	+/1.74	+/1:160
SH21	None	17/M	Direct/seasonal	Yes	None	Seminomadic	80	–/0.36	–/<1:10
SH65	None	20/M	D/daily	No	None	Seminomadic	65	+/1.13	+/1:80
SH43	None	34/M	Direct	No	None	Sedentary	1,600	–/0.76	+/1:160
SH44	None	40/M	Direct	Yes	None	Sedentary	89	–/0.48	–/<1:10

*All 12 patients tested positive by PRNT_50_. IFA, immunofluorescence assay; HPT, hypertension; PRNT_50_, 50% reduction plaque reduction neutralization assay. †Direct indicates camel herders with direct camel contact but extent of exposure is not known; direct/daily, camel herders with daily direct camel contact; direct/monthly, camel herders with monthly direct camel contact; direct/seasonal, camel herders with seasonal direct camel contact; indirect, family members of camel herders. ‡Positive result is >1.1; borderline, 0.8–1.1; negative, <0.8, as defined by the test manufacturer. §Negative test result is <1:10, as defined by the test manufacturer.

All but 2 of the study participants were exposed to camels. There was no significant correlation (p>0.5) between MERS-CoV seropositivity and lifestyle, presence of concurrent conditions, drinking unpasteurized camel milk, or tobacco use, with the caveat that the sample size was small.

## Conclusions

In general, nomads had the most and sedentary populations had the least camel contact, although nearly all family members were exposed to and took care of camels. Of 100 participants, we identified 12 who were MERS-CoV seropositive, as measured by the presence of PRNT_50_ antibody. Of note, several PRNT_50_-positive samples were negative by ELISA, but most were positive by immunofluorescence assay. This lack of concordance between ELISA and PRNT_50_ titers was observed previously (*3*,*11*) and may reflect lower sensitivity of the commercial ELISA kit (*12*). Other coronaviruses circulate in camel populations (*13*), and it is conceivable that the high rate of ELISA seropositivity resulted from immune responses to other, possibly MERS-like, coronaviruses present in Pakistan. Thus, it will be important to assess camel (and human) populations for other coronaviruses that might elicit a cross-reactive response. 

The mechanism of MERS-CoV transmission from camels to humans in Pakistan is not established, but most camel handlers and their families drink fresh camel milk, obtained after young camels have finished nursing. Juvenile camels demonstrate the highest rate of seroconversion and of MERS-CoV positivity (*6*,*14*), so it is possible that drinking fresh milk is a source of infection. In this region of Pakistan, camel handlers and their families also share water sources with camels, which probably contributes to virus transmission. Zohaib et al. identified a 75.6% MERS seroprevalence in camels throughout Pakistan, but 0% seropositivity in humans, including some with camel contact (*7*). 

Medical services in Cholistan and adjacent areas are limited, making MERS diagnosis and transmission studies difficult. Our findings show a need for additional studies to confirm the absence of clinically apparent MERS in this region and to determine whether epidemiologic, technical, or other factors caused differences in seropositivity between our study and that of Zohaib et al. 

Our study, by demonstrating a low but detectable rate of MERS-CoV seropositivity in camel handlers and their families, indicates that this population could contribute to MERS-CoV transmission to the broader community in Pakistan. We previously showed that measurement of T cell responses identified additional MERS-CoV–immune persons (*3*,*11*), suggesting that our results may underestimate the prevalence of MERS-CoV infection. Our results also illustrate the importance of educating camel herders and their families about proper infection control measures, including handwashing, to diminish the likelihood of MERS-CoV transmission.

Appendix Additional information about Middle East respiratory syndrome coronavirus seropositivity in camel handlers and their families, Pakistan.
