# Supplementary material for: Middle East Respiratory Syndrome Coronavirus Seropositivity in Camel Handlers and Their Families, Pakistan
Source: Emerg Infect Dis. 2019 Dec;25(12):2307–9. doi: 10.3201/eid2512.191169 (PMC6874235; doi:10.3201/eid2512.191169)
Supplement: Appendix — Additional information about Middle East respiratory syndrome coronavirus seropositivity in camel handlers and their families, Pakistan. [file 19-1169-Techapp-s1.pdf]

Article DOI: https://doi.org/10.3201/eid2512.191169

Middle East Respiratory Syndrome Coronavirus Seropositivity in Camel Handlers and Their Families, Pakistan

Appendix

Virus and Cells

The EMC/2012 strain of MERS-CoV was provided by Bart Haagmans and Ron Fouchier (Erasmus Medical Center, Rotterdam, the Netherlands). We conducted all work with infectious MERS-CoV in the University of Iowa Biosafety Level 3 (BSL3) Laboratory.

MERS-CoV ELISA

We performed ELISAs, which use the S1 protein as target, as described previously (*11*), using commercially available kits (Euroimmun Medizinische Labordiagnotika AG; https://www.euroimmun.com) and read them as positive (>1.1), negative (<0.8), or borderline (0.8–1.1).

We performed MERS-CoV IFA, which uses MERS-CoV infected cells, as previously described (*11*). We considered titers <1:10 negative.

Plaque Reduction Neutralization Assays ($PRNT_{50}$)

We performed $PRNT_{50}$ assays as previously described (*11*). We repeated assays for $PRNT_{50}$ $\geq$2 times for each serum sample, with nearly identical results. We considered serum samples with $PRNT_{50}$>1:20 positive.

Statistical Analysis

Fisher exact test was used to compare differences between groups. We considered p values <0.05 statistically significant.
